# The kidney in genetic metabolic disorders

**DOI:** 10.1515/medgen-2025-2044

**Published:** 2026-02-18

**Authors:** Ulla T. Schultheiss, Anke Schumann

**Affiliations:** University of Freiburg Department of Medicine IV – Nephrology and Primary Care Hugstetter Str. 49 79106 Freiburg Germany; University of Freiburg Department of Pediatrics, Adolescent Medicine and Neonatology Breisacher Str. 62 79106 Freiburg Germany

**Keywords:** inborn errors of metabolism, monogenic metabolic kidney diseases, Fabry disease, Glycogen storage disease, organic acidurias

## Abstract

Genetic metabolic kidney diseases arise from (likely) pathogenic variants affecting kidney metabolism, causing progressive kidney dysfunction. Symptoms include but are not restricted to nephrolithiasis, proteinuria, kidney failure, and extrarenal manifestations. Genetic testing in combination with metabolic profiling aids early diagnosis and personalized management strategies, which may include enzyme replacement, dietary changes, and kidney-related therapies. Advances in gene therapy and precision medicine offer hope for better outcomes. Early diagnosis and intervention are key to improving prognosis and quality of life, emphasizing the importance of advancing combined metabolic/genetic testing and treatment approaches.

## 1 Introduction – The central role of the kidney for health and homeostasis

The kidney is crucial for homeostasis, filtration, and waste excretion, functions that rely on its complex structure. Disruption of the nephron, the kidney’s functional unit, and its (sub)cellular compartments underlies many forms of chronic kidney disease (CKD). CKD is defined as structural or functional kidney abnormalities, such as albuminuria, hematuria, electrolyte disorders, histological or imaging findings, history of kidney transplantation, or reduced GFR (<60 ml/min per 1.73 m²), that persist for at least 3 months and carry health implications. CKD is classified by cause, GFR category, and albuminuria category, with GFR estimated from serum creatinine for clinical utility, and determining etiology remains essential for personalized management [1]. Causes include common conditions such as hypertension and diabetes mellitus, and rare inherited disorders. Globally, CKD affect 10–13 % of the population [2]. The international classification of inherited metabolic disorders (IMD) comprises ~1450 monogenic disorders [3], over 10 % of which involve the kidney, highlighting the kidney as a target organ in the field of inborn errors of metabolism (IEM). An overview of genetic traits, mechanisms, kidney involvement, extrarenal symptoms, and clinical signs is provided in **Table 1**.

**Figure 1:**
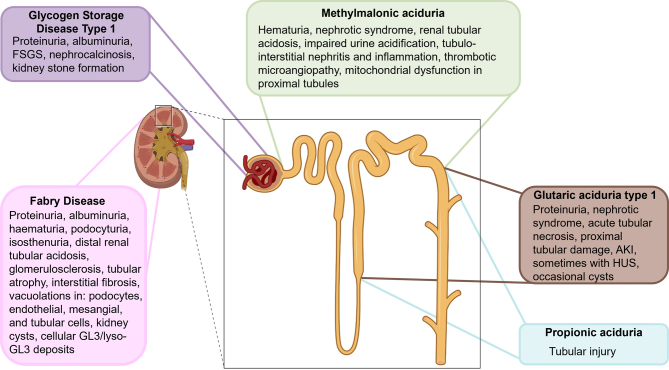
Location of exemplary IEM in comparison to disease manifestations within the kidney. **Legend**: IEM can affect all parts of the kidney; Fabry Disease=all kidney compartments; Glycogen Storage Disease Type 1=primary glomerular disease; Methylmalonic aciduria=proximal and distal tubules; Propionic aciduria and Glutaric aciduria type 1= primarily distal tubule. Created in BioRender, modified in Microsoft PowerPoint. Schultheiss, U. T. (2025) https://BioRender.com/ope56nm

Kidney involvement in IEM can affect all nephron segments, but the patterns of injury differ (**Figure 1**). Glomerular involvement typically presents with albuminuria, whereas distal tubular defects often show no specific patterns. Proximal tubular dysfunction, such as renal Fanconi syndrome in Dent’s disease (*CLCN5*, *OCRL*), is more characteristic, and renal tubular acidosis can occur in organic acidurias. Tubular CKD is challenging to detect due to nonspecific kidney changes. Monitoring is complicated: creatinine is influenced by muscle mass, and cystatin C by inflammation or thyroid function. Moreover, estimating GFR from these markers may remain normal even when significant kidney function is lost. Importantly, eGFR based on creatinine and cystatin C reflects glomerular filtration but does not capture tubular mass, potentially underestimating tubular dysfunction. Estimation equations are also population-specific, so their use should be carefully considered based on the patient group being evaluated.

As a result, injury may manifest as glomerular disease, proximal and distal tubular damage, kidney cysts, nephrocalcinosis, stone formation, and structural anomalies. Diagnosing CKD in the context of IEM is challenging due to multisystem involvement and often late clinical presentation. This review focuses on kidney-related IEMs that contribute to the increasing global CKD burden, characterized by high mortality and treatment costs, underscoring the need for earlier detection and targeted therapies [4] (**Table 1**).

## 2 Glomerular disease in IEM

Primary glomerular disease is characterized by albuminuria, haematuria and impairment of kidney function. Light microscopy of urine can reveal dysmorphic erythrocytes. The clinical spectrum is a continuum and ranges from asymptomatic patients to full-blown nephrotic syndrome (protein excretion > 3500 mg/24h, hypalbuminaemia, hyperlipidaemia) which is a potentially life-threatening complication. A kidney biopsy can help to classify the disease phenotype. Fabry Disease may present with a primary glomerular phenotype, but affects many different parts of the kidney.

### Fabry disease:

**Figure 2:**
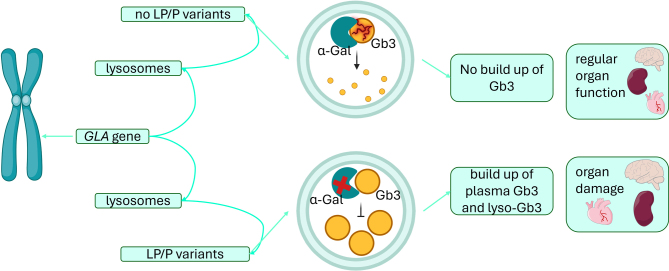
Schematic of *GLA* variants and Gb3 accumulation in Fabry disease. **Legend**: *GLA* variants impair lysosomal function, causing Gb3 and lyso-Gb3 accumulation and organ damage. Created in BioRender and modified by Schultheiss, U. T. (2025) https://BioRender.com/yicy1wa

Fabry disease [5] (FD) is a rare, lysosomal storage disorder caused by (likely) pathogenic variants in the *GLA* gene, causing alpha-galactosidase A (α-Gal A) deficiency and multisystemic accumulation of glycosphingolipids (mainly Gb3 and lyso-Gb3) in various tissues, including the kidneys, heart, and nervous system [5], [6], [7] (**Figure 2**). FD follows an X-linked inheritance pattern. Epidemiologically, the prevalence in white male populations is approximately 1:17,000 to 1:117 000 [8] (**Table 2**). FD is classified into the classic (no enzyme activity, early-onset, multisystem involvement) and the late-onset type (residual enzyme activity) representing distinct clinical phenotypes.

The FD pathogenesis involves lysosomal dysfunction, vascular abnormalities, inflammatory responses, and cellular stress, contributing to multisystem damage [9] (**Figure 2**). Gb3 accumulation disrupts autophagy, causing build-up of dysfunctional components [10], [11], while lyso-Gb3 impairs calcium signaling [12], triggering oxidative stress, mitochondrial dysfunction, and altered gene expression[9]. This leads to early proteinuria and polyuria [13], indicative of glomerular and tubular damage (**Figure 1**). Progressive kidney damage includes glomerulosclerosis [14], endothelial dysfunction, fibrosis, and inflammation [15],[16]. FD is caused by over 1,000 different mostly private variants in the *GLA* gene [17]. The most common variants are missense variants (late-onset of FD) [18]. Nonsense variants, frameshift variants and large deletions result in truncated enzymes (classic type of FD), while splice site variants produce a partially functional enzyme [19]. Some common variants present with well-established genotype-phenotype correlations (e.g., p.Arg301Gln and p.Trp64Ser (classic phenotype [20]). Due to X-inactivation, genotype-phenotype correlations are more difficult to predict in women [21]. Early diagnosis is crucial, since the prognosis is related to early treatment of FD complications [5], [18]. Biochemical testing in males reveals markedly reduced α-Gal A activity, <1 % in classic and >1 % in late-onset forms. Enzyme activity is not reliable in females, necessitating molecular genetic testing to identify a (likely) pathogenic variant in the *GLA* gene. Elevated plasma lyso-Gb3 levels may support diagnosis [5]. The standard treatment for FD is enzyme replacement therapy (ERT), but it requires lifelong infusions and may trigger anti-drug antibodies reducing efficacy [17]. Personalized therapy approaches, such as pharmacological chaperone therapy have emerged. Migalastat, a chaperone that improves protein trafficking, restores enzyme function in 35–50 % of patients with amenable *GLA* variants and shows comparable efficacy to ERT [22]. Gene therapy approaches include non-viral and viral delivery systems [23], solid lipid nanoparticles carrying *GLA* plasmid increase kidney α-Gal A activity more than naked DNA after IV administration [24]. Among viral vectors, adeno-associated viruses (AAVs) are most promising. AAV6.2 and AAV8 enhance kidney targeting via subcapsular injection [25], while AAV9 is optimal for kidney vein delivery [26]. Systemic α-Gal A secretion via AAV also benefits kidneys without direct targeting. A preliminary *ex vivo* lentiviral gene therapy trial in five men with classic FD showed durable leukocyte α-Gal A correction and glycosphingolipid stabilization, allowing 60 % participants to discontinue ERT [27]. Without treatment, male individuals with FD typically have a median lifespan of about 50 years, primarily due to cardiovascular and kidney complications. ERT, particularly when started early, can enhance organ function and increase life expectancy making early recognition paramount for improving prognosis. While Fabry disease exemplifies a lysosomal storage disorder with prominent glomerular involvement, other metabolic defects affect kidney function through different mechanisms. One such example is glycogen storage disease type 1, where impaired glucose metabolism plays a central role.

**Table 1:** Summary of genetic traits, mechanisms, kidney involvement, extrarenal symptoms, clinical signs for FD, GSD1, DD, MMA-uria, PA-uria, and GA1

**Disease**	**Genetic traits**	**Mechanism**	**Kidney involvement (frequency & importance)**	**Extrarenal symptoms (frequency & importance)**	**Clinically dominant features**
FD	XL; *GLA*	Lysosomal storage disorder; Gb3/lyso-Gb3 accumulation → lysosomal dysfunction, vascular injury	Very frequent: early proteinuria, polyuria, CKD progression (common in males, variable in females). Kidney disease is a major cause of morbidity and mortality.	Almost universal systemic disease: neuropathic pain, angiokeratomas, cardiac disease (LVH, arrhythmias), stroke.	Extrarenal disease (cardiac & neurologic) usually dominates survival prognosis, but kidney involvement drives morbidity and ERT eligibility.
GSD1a/b	AR; *G6PC* (1a), *SLC37A4* (1b)	Defective glycogenolysis → glycogen accumulation	Common: albuminuria, tubular acidosis, nephrolithiasis; CKD prevalence ~30–40 % in adults, but progression is milder with modern therapy. Historically severe.	Universal: hypoglycemia, hepatomegaly, metabolic crises. GSD1b adds neutropenia/infections.	Metabolic instability and liver disease dominate in childhood; kidney disease is important for long-term adult prognosis.
DD	XL; *CLCN5* (DD1), *OCRL* (DD2)	Proximal tubular endocytosis defect → LMW proteinuria, impaired receptor trafficking, autophagy stress	Universal in affected males: LMW proteinuria present in nearly all; CKD progression to KF in 30–50 years is common.	Rare extrarenal signs (in DD2: mild cataracts, short stature, mild intellectual disability).	Kidney disease dominates (proteinuria, nephrolithiasis, CKD). Extrarenal features minor.
MMA-uria	AR; *MUT*, cobalamin disorders (*MMAA*, *MMAB*, *MMADHC*)	Defective propionate metabolism → accumulation of characteristic organic acids (MMA in particular)/ acylcarnitines, secondary mitochondrial dysfunction	Very common: CKD in up to 60 % of children (median onset ~6–11 yrs). Kidney dysfunction is a major long-term complication.	Frequent and severe: metabolic crises, developmental delay, failure to thrive, neurologic involvement.	Both extrarenal metabolic crises (life-threatening) and progressive CKD are clinically important; extrarenal issues dominate early, kidney disease later.
PA-uria	AR; *PCCA*, *PCCB*	Defective propionate metabolism, accumulation of characteristic organic acids / acylcarnitines, secondary mitochondrial dysfunction	Common in adults: ~50 % have CKD (GFR <60 mL/min/1.73 m²). Rare in children. Kidney disease is a late complication.	Frequent and severe: metabolic crises, neurologic symptoms, cardiomyopathy/arrhythmias (a key prognostic factor).	Extrarenal crises & cardiomyopathy dominate prognosis. Kidney disease relevant for long-term survival into adulthood.
GA1	AR; *GCDH*	Impaired lysine degradation → glutaric and 3-OH glutaric acid accumulation, characteristic acylcarnitine profile (C5-DC)	Moderate frequency: ~25 % of adults develop CKD; occasional pediatric AKI (HUS). Not always clinically obvious.	Very frequent & devastating: acute encephalopathic crisis in infancy (0–6 yrs) if untreated→ dystonia, motor disability, developmental regression, insidious onset is reported	Neurologic disease dominates prognosis; kidney involvement is secondary but relevant for long-term adult management.

**Legend:** FD=Fabry Disease, GSD=glycogen storage disease, DD=Dent Disease, MMA-uria=methylmalonic aciduria, PA-uria=propionic aciduria, GA=glutaric aciduria, XL=X-linked mode of inheritance; α-Gal A=alpha-Galactosidase A, Gb3 and lyso-Gb3=glycosphingolipids, CKD=chronic kidney disease, LVH=left ventricular hypertrophy, ERT=enzyme replacement therapy, AR=autosomal recessive mode of inheritance, LMW=low molecular weight, KF=kidney failure, TCA=tricarboxylic acid, AKI=acute kidney injury, HUS=hemolytic uremic syndrome.

### Glycogen storage disease type 1

Glycogen storage disease type 1 (GSD 1) is a rare condition causing disturbances in the break-down of glycogen and glucose generation. The condition is caused by biallelic (likely) pathogenic variants in the *G6PC* gene coding for the hepatic enzyme glucose-6-phosphatase (GSD 1a) or the *SLC37A4* gene coding for the glucose-6-phosphate transporter (GSD 1b) [28]. Epidemiologically, the overall incidence is 1:100 000 [29]. GSD 1 is characterized by accumulation of glycogen and fat particularly in the liver and the kidneys causing organomegaly and metabolic dysfunction (**Figure 1**). The clinical phenotype includes severe hypoglycaemia, lactic acidosis, hyperuricaemia and hypertriglyceridaemia. GSD 1b patients suffer from severe neutropenia in addition. There is no pathognomonic “metabolic” biomarker for GSD 1. Untreated patients present with characteristic stigma as short stature, doll-like face, hepatic adenoma and CKD. Dietary therapy includes frequent meals preferentially using complex carbohydrates and uncooked cornstarch including nighttime dosing to avoid life threatening hypoglycaemia. SGLT2-inhibotors (gliflozins) have been repurposed to treat GSD 1b induced neutropenia. CKD is a common and well-known long-term complication in GSD 1. The kidney phenotype comprises albuminuria, proximal and distal tubular acidosis, nephrolithiasis and finally kidney failure [30]. Kidney stone formation due to hypercalciuria and hyperuricaemia has been reported [31], [32]. Glomerular hyperfilration followed by microalbumunuria is often the presenting sign of CKD [33]. In the few existing human and murine kidney biopsies, focal segmental glomerulosclerosis, tubular atrophy and tubular interstitial nephritis have been reported [34], [35]. In the past, albuminuria progressing to kidney failure commonly occurred in early childhood. With standardized dietary regimen and blood glucose control resulting in improved metabolic stability kidney-related complication have become less frequent [36] (**Table 1**). The early introduction of ACE-inhibitors and consequent treatment of hyperuricaemia have also shown kidney protective effects [37], [38]. CKD still remains a problem in GSD 1: a recent study suggests that 36 % and 13.8 % of patients above 25 years were affected by albuminuria respectively [28] (**Table 2**). Of note, CKD was rather mild and none of the patients (oldest patient 56 years) needed kidney replacement therapy [28]. The pathophysiology of CKD in GSD is not fully understood, several pathways represent potential therapeutic targets: Elevation of ER stress markers and impaired autophagy have been discussed in a GSD 1a mouse model. Increased activity of the pentose phosphate pathway has been shown to alter the NADPH/NADP+ ratio, promote inflammation and induce oxidative stress [39]. Kidney fibrosis in GSD 1 is linked to extracellular matrix protein build-up. SGLT2 inhibitors reduced glycogen and improved kidney structure and function in a murine model for GSD 1b, although long-term clinical benefits remain unclear [40]. Clinical trials phase I/II for both viral (AAV8, NCT03517085) and non-viral (mRNA, NCT05095727) vectors are currently recruiting and/or analyzing first results in pediatric and adult GSD 1a patients [41]. The primary endpoint is metabolic stability (efficacy of increased fasting tolerance) and not CKD prevention, but long-term kidney prognosis remains an important secondary consideration. An overview of phenotype, epidemiology, prognosis, and therapy for GSD1 is provided in **Table 2**. In contrast to the primarily glomerular manifestations seen in Fabry disease and the mixed metabolic–glomerular phenotype of GSD1, certain inborn errors of metabolism predominantly affect the proximal tubule. Dent disease is a key representative of this group.

**Table 2:** Summary of phenotype, epidemiology, prognosis, and therapy of FD, GSD1, DD, MMA-uria, PA-uria, and GA1

**Disease**	**Phenotype**	**Epidemiology**	**Prognosis**	**Therapy**
Fabry Disease (FD)	Classic: multisystem involvement (kidneys, heart, CNS), early-onset. Late-onset: residual enzyme activity, less severe.	X-linked; prevalence ~1:17,000–1:117 000 in white males	Without treatment: median lifespan ~50 years, mainly due to cardiac & kidney complications. Early therapy improves organ function and life expectancy.	Enzyme replacement therapy (ERT), pharmacological chaperone (migalastat), emerging gene therapy (AAV, lentiviral).
Glycogen Storage Disease Type 1 (GSD1)	Hypoglycemia, lactic acidosis, hyperuricemia, hypertriglyceridemia; Hepatomegaly, nephromegaly, liver adenomas, hepatocellular carcinomas; short stature, doll-like face; GSD1b: neutropenia	Rare; incidence ~1:100 000	CKD common; albuminuria in adults (36 %/13.8 %); early metabolic control improves long-term prognosis	Dietary therapy (frequent meals, complex carbs, uncooked cornstarch), SGLT2 inhibitors for neutropenia, ACE-inhibitors for CKD, experimental gene therapy (viral/non-viral).
Dent Disease (DD)	Proximal tubular dysfunction, LMW proteinuria, hypercalciuria, nephrocalcinosis, sometimes FSGS; variable in females	X-linked; prevalence 1:400 000–1:1,000 000	Many develop CKD stage 5 between ages 30–50; severity variable	Supportive care, CKD management; kidney transplantation curative; genetic counseling for families.
Methylmalonic Aciduria (MMA-uria)	Metabolic crisis (lactic acidosis, hyperammonemia), failure to thrive, developmental delay, movement disorders, CKD	<1:100 000 in NA, Europe, Asia-Pacific; higher in Middle East, North Africa, Japan	CKD develops in up to 60 % of children (median onset 6–11 y); severity depends on residual MCM activity	Protein restriction, precursor-free amino acids, carnitine, hydroxycobalamin (if responsive), organ transplantation (kidney/liver/combined), experimental mRNA therapy (mRNA-3705).
Propionic Aciduria (PA-uria)	Similar to MMA; metabolic crises, failure to thrive, seizures, cardiomyopathy; CKD appears mainly in adults	0.33:100 000 newborns in Europe	Adult CKD common; GFR <60 mL/min/1.73 m² in ~50 % of adults; variable course	Protein restriction, precursor-free amino acids, carnitine; organ transplantation (kidney/liver/combined); experimental mRNA therapy (mRNA-3927).
Glutaric Aciduria Type 1 (GA1)	Acute metabolic crisis in infancy, striatal injury → dystonia; kidney involvement: occasional CKD, tubular changes in animal models	1:100 000	Early detection & metabolic control improves neurological prognosis; CKD occurs in ~25 % of adults	Low-protein diet, precursor-free amino acids, carnitine supplementation; early treatment crucial; intrathecal gene therapy (VGM-R02b) under investigation.

**Legend:** CNS=central nervous system, CKD=chronic kidney disease, SGLT2-inhibitors=sodium-glucose cotranstranporter-2 inhibitors, ACE-inhibitors=angiotensin-converting enzyme inhibitors, mRNA=messenger ribonucleic acid

## 3 Proximal tubular involvement in IEMs

IEMs involving the proximal tubule are characterized by impaired solute reabsorption and disruptions in cellular metabolism. These conditions often cause proximal tubulopathies, with urinary loss of glucose, amino acids, phosphate, bicarbonate, and low-molecular-weight proteins. A hallmark of these disorders is mitochondrial dysfunction in proximal tubular epithelial cells, which are highly energy-dependent due to their active transport roles. Metabolic stress and DNA damage in these cells can trigger systemic effects and contribute to progressive CKD. Over time, isolated tubular defects may evolve into kidney failure. Disorders such as Dent disease serve as illustrative examples.

### Dent disease

Dent disease (DD) is a rare, X-linked inherited tubulopathy, characterized by proximal tubular dysfunction, low-molecular-weight proteinuria (LMWP), hypercalciuria, nephrocalcinosis, and progressive CKD. DD leads to tubular proteinuria without typical signs of nephrotic syndrome, resulting in possible underrecognition and/or diagnostic delay (**Table 2**). Epidemiologically, the prevalence of DD is estimated between 1 in 400 000 to 1 in 1,000 000. Most affected individuals are male, and female carriers may exhibit mild features such as hypercalciuria or proteinuria, occasionally developing nephrocalcinosis or CKD later in life (**Figure 1**). DD is classified into two major types: DD type 1: Caused by (likely) pathogenic variants in the *CLCN5* gene (50–60 %), which encodes the ClC-5 chloride/proton exchanger [42]. DD type 2: Caused by (likely) pathogenic variants in the *OCRL* gene (15 %), also involved in Lowe syndrome [43]. 25–35 % of DD patients have no identified variant, suggesting additional genetic contributors [44]. *De novo* variants are found in about 12 % of DD1 and up to 30 % of DD2 cases [44]. Both ClC-5 and OCRL dysfunctions cause failure in receptor-mediated endocytosis of LMW proteins in proximal tubular cells [44]. This leads to low-molecular-weight proteinuria [44]. ClC-5 is primarily expressed in early endosomes, and its dysfunction results in abnormal trafficking and reduced expression of endocytic receptors [45]. Similarly, OCRL dysfunction increases PI(4,5)P2 levels, disrupting actin dynamics and endosomal function, causing impaired reabsorption and autophagic stress [44]. In both DD1 and DD2, secondary mechanisms, including tubulointerstitial inflammation, autophagy dysregulation, and potential podocyte injury, may contribute to progressive kidney fibrosis and CKD [46]. ClC-5 and OCRL have also been detected in podocytes, potentially explaining cases of focal segmental glomerulosclerosis (FSGS) on biopsy [47]. Over 300 distinct (likely) pathogenic variants in *CLCN5* have been reported, including truncating, missense, and splice variants [48]. Though functional studies show varying effects, clear genotype–phenotype correlations are limited [44]. Some data suggest truncating variants or (likely) pathogenic variants affecting the ClC-5 pore domain may associate with earlier CKD [49]. For *OCRL*, DD2-related variants cluster in exons 1–7, while those in exons 8–24 tend to cause full Lowe syndrome, phenotypic overlap exists [50]. Symptoms begin in childhood but may remain unrecognized [44]. Proteinuria is present in virtually all cases and may reach nephrotic-range, although serum albumin levels are normal. Hypercalciuria (~61-90 % of cases), is age-dependent and may diminish with progressive CKD. Nephrolithiasis and nephrocalcinosis are common complications, along with rickets and growth retardation. Proximal tubulopathy may occur, though full Fanconi syndrome is rare. In DD2, mild extrarenal features (congenital cataracts, short stature, mild intellectual disability) may occur (**Table 1**). Many patients reach CKD stage 5 between ages 30 and 50. Disease severity varies, even within families. Early diagnosis is critical but often missed. Key diagnostic steps include: Measurement of low-molecular-weight proteinuria (e.g., α1-microglobulin, retinol-binding protein); urine calcium/creatinine ratio for hypercalciuria; genetic testing for *CLCN5* and *OCRL* to confirm diagnosis and determine type. Differential diagnoses include other genetic proximal tubulopathies (e.g., cystinosis), acquired conditions (e.g., drug toxicity), and glomerular diseases. Treatment is exclusively supportive. Kidney transplantation is curative and recurrence does not occur; female relatives need careful evaluation as potential donors. Kidney failure is common, with no strong correlation between proteinuria severity and eGFR decline. Nephrocalcinosis does not predict disease progression. Early diagnosis, genetic confirmation, and supportive care can delay CKD progression and prognosis (**Table 2**). Ongoing care should be coordinated through tertiary nephrology centers with DD expertise. Genetic counselling in at-risk families is advised. Targeted supportive therapy remains central to management. While Dent disease highlights disorders of the proximal tubule, metabolic conditions can also compromise the distal tubule. Organic acidurias serve as an illustrative group, where the exact pathomechanism of CKD remains unclear and a typical CKD pattern (like e.g. proteinuria) is lacking, although rare biopsies suggest that the tubular compartment is more affected than other compartments.

## 4 Distal tubular involvement in IEM

The kidney is frequently affected in IEM, with both glomerular and tubular compartments involved. Glomerular injury often presents with albuminuria, while distal tubular involvement, as seen in organic acidurias (OAs), typically lacks a specific diagnostic pattern and may be subtle. Different pathomechanisms (e.g. mitochondrial dysfunction, accumulation of potentially toxic organic acids, …) contributing to tubular injury are discussed and CKD in OAs is difficult to detect. Early detection of CKD requires, besides creatinine and cystatin C, the investigation of urinary biomarkers of tubular dysfunction (e.g., low-molecular-weight proteins), functional assessments, and careful clinical monitoring of electrolytes and acid-base status. Combining these approaches can help identify subtle tubular injury before overt kidney impairment develops and understand disease causing patterns in the future.

### Organic acidurias

OAs comprise a group of IEM involved in the break-down of branched chain amino acids and CoA-activated carboxylic acids. Deficiencies of the respective enzymes cause accumulation of various metabolites some of which interfere with mitochondrial energy generation via disturbances of the tricarboxylic citric acid cycle or the urea cycle. Three OAs are of particular importance and have been associated with CKD development. These are Methylmalonic- (MMA-uria), propionic (PA-uria) and glutaric aciduria type 1 (GA-uria) [51], [52]. All conditions follow autosomal recessive inheritance.

### Methylmalonic aciduria

Classical MMA-uria is caused by (likely) pathogenic variants in the *MUT* gene coding for methylmalonyl CoA mutase (MCM). MCM depends on vitamin B_12_ (=5-deoxy-adenosyl-cobalamin) as essential cofactor. Deficiencies in the transport or synthesis cobalamin (*MMAA, MMAB, MMAD)* and methylmalonyl-CoA epimerase deficiency (*MCEE*) also cause isolated MMA-uria. Epidemiologically, the incidence of isolated MMA-uria is less than 1:100 000 in North America, Europe, and the Asia-Pacific region, whereas higher rates were observed in the Middle East, North Africa, and Japan [53]. MCM is involved in the break-down of certain amino acids (isoleucine, methionine, threonine, valine, odd-chain fatty acids and cholesterol). Metabolic profiling reveals elevated levels of disease-specific organic acids such as methylmalonic acid (MMA), 3-hydroxypropionate and 2-methylcitrate. Plasma amino acid analysis often shows elevated glycine and occasionally alanine. The acylcarnitine profile shows increased propionylcarnitine (C3) and variable elevations in methylmalonic/succinylcarnitine (C4DC) [53]. Patients are at risk of acute life-threatening metabolic crisis including (lactic) acidosis and hyperammonaemia. Long-term complications comprise failure to thrive, developmental delay, movement disorders and CKD. Disease and CKD severity is associated with residual MCM activity (Mut^0^, Mut^-^) and vitamin B_12_ responsiveness (Mut- and CblA/B/D), which inversely correlates with the height of MMA [54]. The therapy consists of natural protein restriction, supplementation with precursor-free amino acid mixtures, carnitine and i.m. administration of hydroxycobalamin in case of cofactor responsiveness. Organ transplantation (both liver and kidney) can be performed to improve metabolic stability and correct for kidney failure. Liver and liver/kidney transplantations significantly reduce MMA levels and lead to kidney function preservation in a 2-year observational study [55]. All isolated MMA-uria patients are at risk for CKD development. Up to 60 % of children with MMA-uria suffer from CKD with a median age of onset between 6-11 years [56], [57]. Tubulointerstitial nephritis with mononuclear infiltrations has been reported in kidney biopsies. Renal tubular dysfunction in MMA-uria patients is not a consistent finding (**Figure 1**). Kidney disease is thought to result from secondary mitochondrial dysfunction rather than from the direct nephrotoxic effects of MMA or other toxic metabolites [58], [59]. Alterations of mitochondrial dynamics and homeostasis and disturbance of both mito- and autophagy have been reported [60], [61] as potentially disease-driving mechanisms (**Table 1**). An international study assessing safety, pharmacokinetics, and treatment pharmacodynamics of mRNA-3705 for isolated MMA-uria is recruiting (NCT04899310). Closely related to MMA both genetically and biochemically, propionic aciduria involves a defect one step upstream in propionate metabolism, resulting in a comparable but distinct pattern of systemic and kidney disease.

## Propionic aciduria

Propionic aciduria (PA) is caused by (likely) pathogenic variants in the *PCCA* or *PCCB* gene. The genes code for the alpha- and beta-subunit of propionyl CoA carboxylase (PCC), an enzyme localized one step up-stream of MCM. PCC catalyzes the conversion of propionyl-CoA to methylmalonyl-CoA fuelling the TCA-cycle with succinyl-CoA for energy generation. Epidemiologically, the estimated incidence of PA-uria is 0.33:100 000 newborns in Europe [62]. The spectrum of disease-specific, accumulating metabolites is very similar to MMA-uria, with the exception of methylmalonic acid and methylmalonyl carnitine. Therapy options are comparable to MMA-uria. Life-threatening, acute metabolic crisis is a life-long risk and triggered by catabolic states, which are often associated with infections and/or reduced calory intake. As in MMA-uria, protein overload is an additional risk. Long-term complications comprise failure to thrive, developmental delay, seizures [63] and cardiac complications: Cardiomyopathy and arrhythmia (long QT syndrome) are a common complications in PA-uria [64]. Unlike MMA-uria, CKD in PA-uria typically appears in adulthood. In a cohort of 31 PA-patients, half of the adult patients showed a significant kidney function decline (GFR < 60 mL/min/1.73m^2^) [65]. Creatinine-based GFR poorly predicts kidney function in these patients due to protein-restricted diets. Like MMA-uria, mitochondrial dysfunction is suspected to underlie CKD here [66]. Studies in human kidney cells and a murine PA model support disturbed mitochondrial homeostasis as causal of CKD. Human kidney biopsy data in PA-uria revealed enlarged mitochondria with disrupted cristae, tubular cell vacuolization (**Figure 1**), focal dilation, and flattened epithelium [67]. No CKD-specific phenotype (e.g. proximal tubular dysfunction, albuminuria) was detectable. Organ transplantation (kidney, liver, combined) may improve metabolic stability and organ function. An international open label study of mRNA-3927 treatment in PA-uria is currently recruiting (NCT04159103). Beyond propionate metabolism, glutaric aciduria type 1 illustrates how impaired lysine degradation contributes to kidney injury, adding another dimension to the spectrum of organic acidurias with kidney involvement.

## Glutaric aciduria type 1

Glutaric aciduria type 1 (GA1) is caused by biallelic (likely) pathogenic variants in the *GCDH* gene coding for the mitochondrial enzyme glutaryl-CoA dehydrogenase. Epidemiologically, the incidence is 1:100 000. The enzyme is involved in the break-down of lysine, hydroxylysine and tryptophane. Glutaric acid, 3-OH-glutaric acid and glutarylcarnitine (C5-DC) are biochemical hallmarks of GA1 [52]. Untreated patients suffer from acute encephalopathic metabolic crisis, which classically occurs in a vulnerable window between 0 and 6 years of life. Severe dystonia due to striatal injury is the devastating consequence and associated with high morbidity and mortality. A low protein diet, precursor free amino acid mixtures and carnitine therapy to facilitate the excretion of toxic metabolites are advised, but can be eased with age. Newborn screening and subsequently early therapy dramatically improved patients’ prognosis. As for MMA- and PA-uria, catabolism and/or protein overload is the trigger for metabolic decompensation [52]. There are case reports on acute kidney injury in pediatric GA1, mostly haemolytic uremic syndrome (HUS; n=2) [68], [69] but also one case of nephrotic syndrome [70]**.** Given these kidney complications occur relatively frequently in the general pediatric population, a coincidental association with GA1 cannot be entirely excluded. Investigation of a huge European study cohort revealed CKD (defined as GFR < 60 ml/min/1.73 m²) in 25 % of all adult GA1 patients (**Table 2**). Differently to MMA-uria, high excretors have not been affected more frequently or severely than low excretors [71]. Animal models of GA1 showed kidney involvement: GA1 mice on a high-protein diet exhibited structural and functional kidney changes, including reduced brush border membrane thickness and mitochondrial swelling in proximal tubular cells. Additionally, mRNA expression of organic anion transporters (*Oat1*, *Oat2*) was reduced [72]. Data from a rat *Gcdh*^ki/ki^ model reproduced GFR decline with age. Histopathology revealed dilation of kidney tubules and mononuclear infiltrations. Metabolic stress imposed by a high lysine diet caused early GFR decline and tubulopathy in young rats of this model [73]. To the best of our knowledge, there is no report on human kidney biopsy in GA1 (except [68]), where HUS of unclear aetiology was suspected. A phase 1 study is ongoing for intrathecal gene replacement therapy (VGM-R02b) targeting the *GCDH-gene*. In summary, OAs are rare but relevant conditions associated with CKD in the field of IEM. Good metabolic control does not prevent CKD progression and treatment is exclusively symptomatic. There is no pathognomonic kidney pattern. Deep clinical phenotyping and basic research are required to finally unravel the underlying pathomechanisms and causally treat CKD in these disorders.

## Conclusion

Genetic metabolic kidney diseases are a diverse group of rare disorders often underdiagnosed despite their considerable impact on kidney health. Both **Table 1** and **Table 2** provide structured overviews of disease-specific and mechanistic insights to support clinical interpretation. Key pathological features include mitochondrial dysfunction, impaired solute transport, and systemic metabolic stress. Although ERT and emerging gene therapies have advanced treatment, early diagnosis remains challenging due to variable symptoms and lack of specific kidney biomarkers. Conventional kidney tests often miss early disease, delaying intervention. Recent progress in genomics, screening, and translational research is improving diagnosis and enabling personalized therapies (mRNA treatments, gene delivery, pharmacological chaperones), when started early. To fully realize these opportunities, increased clinician awareness, and integration of genetic diagnostics into nephrology care pathways are essential. Deeper genotype–phenotype insights, prospective studies, and international collaboration will be key to improving outcomes.
